# Prevalence of oral health problems in a Norwegian older adult population: The HUNT Study

**DOI:** 10.1186/s12903-025-06683-y

**Published:** 2025-09-30

**Authors:** Sandra Helena Cetrelli, Yi-Qian Sun, Wenche Moe Thorstensen, Hedda Høvik, Linda Gjøra, Lars Martin Berg, Håvard Kjesbu Skjellegrind, Tone Natland Fagerhaug, Ingvild Paur, Marit Kolberg

**Affiliations:** 1 Department of Neuromedicine and Movement Science, Norwegian University of Science and Technology, Trondheim, Norway; 2 Center for Oral Health Services and Research Mid-Norway (TkMidt), Trondheim, Norway; 3 Department of Clinical and Molecular Medicine, Norwegian University of Science and Technology, Trondheim, Norway; 4 Department of Pathology, Clinic of Laboratory Medicine, St. Olavs Hospital, Trondheim University Hospital, Trondheim, Norway; 5 Department of Otolaryngology, Head and Neck Surgery, St. Olavs Hospital, Trondheim University Hospital, Trondheim, Norway; 6 The Norwegian National Centre for Ageing and Health, Vestfold Hospital Trust, Tønsberg, Norway; 7 Department of Psychiatry, Levanger Hospital, Nord-Trøndelag Hospital Trust, Levanger, Norway; 8 Public Dental Health Services, Trøndelag County, Norway; 9 Department of Clinical Dentistry, UiT The Arctic University of Norway, Tromsø, Norway; 10 HUNT Research Centre, Department of Public Health and Nursing, Norwegian University of Science and Technology, Levanger, Norway; 11 Levanger Hospital, Nord-Trøndelag Hospital Trust, Levanger, Norway; 12 Department of Public Health and Nursing, Norwegian University of Science and Technology, Trondheim, Norway; 13 Norwegian Advisory Unit On Disease-Related Undernutrition, Oslo, Norway; 14 Department of Clinical Service, Division of Cancer Medicine, Oslo University Hospital, Oslo, Norway

**Keywords:** HUNT, HUNT4 70 +, ROAG-J, Oral health, Older adults, Community dwellers, Nursing home residents

## Abstract

**Background:**

Studies show that oral health problems are common among older adults. However, population estimates of oral health problems are largely restricted to individual subgroups. Thus, we aimed to estimate the prevalence of oral health problems in an older adult population comprised of both community dwellers and nursing home residents, and to assess differences between sexes, age groups, and these living situations.

**Methods:**

This cross-sectional study used data from HUNT4 Trondheim 70 + , a part of the fourth Trøndelag Health Study (HUNT4), Norway. Oral health was assessed using the Revised Oral Assessment Guide – Jönköping (ROAG-J). Dental status and use of dentures were evaluated as part of the oral health assessment. A total of 1562 participants aged ≥ 70 years had complete data on ROAG-J and were included for analysis. Prevalence estimates were standardised using inverse probability weighting.

**Results:**

The standardised prevalence of oral health problems and severe oral health problems were 56.1% (95% CI 53.5–58.7) and 9.4% (95% CI 8.0–10.9). The three most common problems were related to teeth (31.9%), dentures (22.0%), and tongue (16.3%). A total of 9.2% were edentulous and 29.6% used full or partial dentures. Differences in prevalences between sexes were found for oral health problems, edentulism, and the use of dentures. The prevalence of having oral health problems, few or no teeth, and using dentures increased with age, and was higher among nursing home residents than community dwellers.

**Conclusion:**

More than half of the population ≥ 70 years of age had oral health problems that indicated the need for preventive measures or further clinical assessment. This was especially apparent among the oldest and nursing home residents.

**Supplementary Information:**

The online version contains supplementary material available at 10.1186/s12903-025-06683-y.

## Background

Maintaining good oral health is important for healthy ageing as it plays a role in multiple aspects of life and function, including the ability to socialise, eat, and quality of life [1, 2]. This is, and will become, increasingly important in a public health perspective as the number of older adults is expected to increase during the upcoming decades [3]. Tooth retention in older age has become evident among adults in high-income countries, indicating an improvement in oral health [4–8]. Despite this, there are still considerable numbers of older adults affected by tooth loss and edentulism in older age [5, 6, 8–10]. Prosthetic treatment such as dentures is therefore common among older adults [9, 11]. However, taking care of ones remaining teeth, dentures, and mouth can become a challenge for some. Factors such as low socioeconomic status, frailty, and cognitive impairment can put older adults at risk of poor oral health [12–14]. Studies show that oral conditions are common in the older adult population [10], with the care-dependent being especially vulnerable to poor oral health as many have unmet oral care needs [15, 16]. This underscores the importance of raised oral health awareness within public health, including primary health care.

To develop and plan sustainable health care services for the older adult population, there is a need for comprehensive descriptive data on oral health. However, few population studies have investigated the prevalence of oral health problems in representative samples of adults 70 years and older, including both community dwellers and nursing home residents. Thus, the overall aim of this study was to estimate the prevalence of oral health problems based on a representative sample of the older adult population in one of Norway’s larger cities. We also explored differences in oral health problems between sexes, age groups, and between community dwellers and nursing home residents.

## Methods

### Study population

**Fig. 1:**
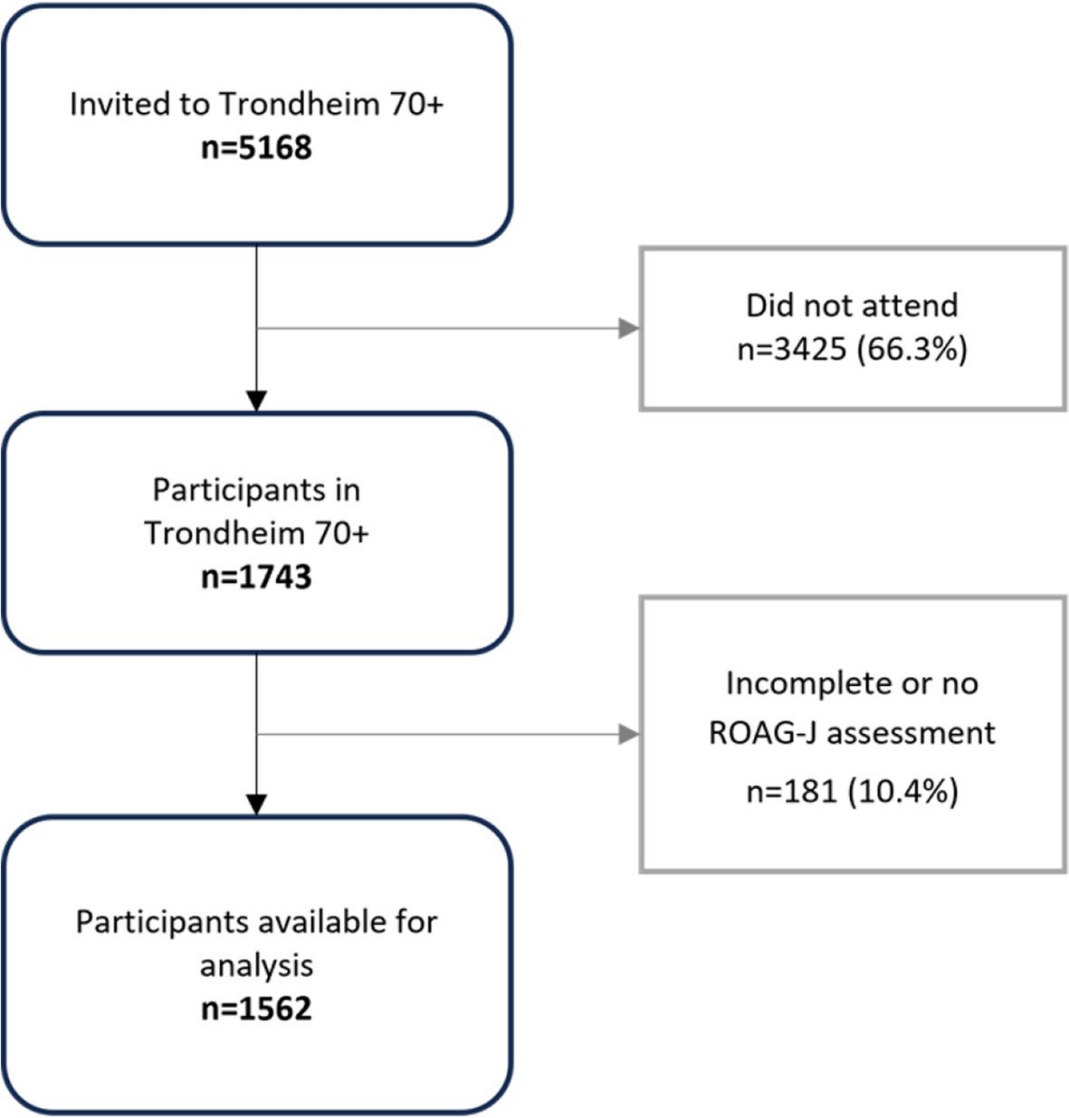
Flow chart of selection process of participants eligible for analysis from HUNT4 Trondheim 70 +

In this cross-sectional study, we investigated data from HUNT4 Trondheim 70 +, an additional part of HUNT4 70 + which refers to the assessments of adults aged ≥ 70 years in the fourth population-based survey of the Trøndelag Health Study (HUNT4) [17]. Trondheim 70 + was conducted in districts of Trondheim Municipality in Central Norway. Among the 5168 eligible participants invited by mail, 1743 (34%) participated from October 2018 to June 2019. Data were collected at three test locations: a field station, in participants’ homes, and nursing homes. Ambulant teams conducted the data collection at the two latter locations. Participants examined at home consisted of individuals receiving home care and individuals not able to attend the field station for other reasons. The personnel at all test locations consisted of health care workers from Trondheim Municipality and nursing students from the Norwegian University of Science and Technology (NTNU). All personnel received practical and theoretical training, and data were collected through self-administered questionnaires, standardised physical examinations, and interviews. The questionnaire and interview data were collected from the HUNT4 Survey [17], and are available through the HUNT Research Centre’s databank website [18]. As part of the physical examination, an oral health assessment was performed at all test locations. In the current study, participants with complete oral health assessments were included for analysis (*n* = 1562). A flow chart of the selection process is shown in Fig. 1.

### Oral health assessment

Oral health was evaluated using the Revised Oral Assessment Guide – Jönköping (ROAG-J) [19]. This standardised version of the screening tool is a further development of ROAG [20], which originate from the Oral Assessment Guide (OAG) by Eilers et al. [21]. When used by trained non-dental health care personnel, ROAG has demonstrated good validity [22]. As a risk assessment tool, ROAG-J is meant to increase awareness as well as systemise oral health care as part of person-centred care [19]. In ROAG-J, nine items including voice, lips, mucus membranes, tongue, gums, teeth, dentures, saliva, and swallowing function are evaluated based on appearance. Each item is given a score of 1–3: score 1) normal or healthy, score 2) a moderate oral health problem (that can be remedied by local health care personnel), and score 3) a severe oral health problem (indicates the need for a referral to a dentist or physician). In addition, score 0 can be assigned to five of the items. For gums, teeth, and dentures, score 0 means the participant has no teeth or no dentures. For voice and swallowing function, a score 0 means it is not applicable to assess [19].

In HUNT4, a Norwegian translation of ROAG-J was used. The personnel performing the ROAG-J assessment were given theoretical and practical training by a dentist or dental hygienist. A picture manual showing examples of the individual items and respective scores was provided to aid the assessments. The equipment used were paper towels, alcohol-based sanitizer, gloves, toothbrush, lubrication (Vaseline), and a flashlight. The item saliva was assessed by sliding the handle of the toothbrush against the buccal mucosa. In the current study, we have presented ROAG-J data in four different ways: 1) Each ROAG-J item with scores presented as they are, excluding score 0. 2) ROAG-J total score, in which the scores from all nine items were summarised (possible range 9 to 27). For this purpose, score 0 was converted to score 1. 3) Oral health problem, defined as having at least one score 2 or 3 for any of the nine ROAG-J items (binary variable). 4) Severe oral health problem, defined as having at least one score 3 for any of the nine ROAG-J items (binary variable).

The oral health assessment also included observations of own teeth (yes/no) and more or fewer than six teeth in the upper and lower jaw. Based on this, we categorised dental status into three groups: more than six teeth in both jaws, six or fewer teeth in one or both jaws, and edentulous. Participants with dentures were registered as having either full or partial dentures (yes/no). Fourteen participants had missing observations of teeth in one or both jaws and were excluded from analyses of dental status and denture use.

### Other variables

Age was used both as a continuous and categorical variable with four categories: 70–74, 75–79, 80–84, and 85 + years of age. Living situation, based on test location, was grouped into community dwellers (combining field station and home visit) and nursing home residents. Highest attained education was obtained from the National Education Database by Statistics Norway [23] and was combined into primary (≤ 10 years) and secondary (11–13 years) school, and college/university (≥ 14 years). Marital status was grouped into unmarried, married, widow(er), and separated/divorced. Smoking habits were grouped into never smoker, previous smoker, and current smoker. Self-perceived health was dichotomised into good (combining good and very good) and poor (combining not so good and poor). Both smoking habits and self-perceived health were collected through self-administered questionnaires. Body mass index (BMI) was calculated as weight in kilograms divided by squared height in metres, based on measurements from Seca 813 scales and Seca 217 stadiometers (Seca, Germany). BMI was used both as a continuous variable, and categorical variable with two categories: < 22 kg/m^2^ and ≥ 22 kg/m^2^, which are recommended age adjusted cut-off in malnutrition-diagnostics [24]. Cognitive function was grouped into no cognitive impairment, mild cognitive impairment (MCI), and dementia. In HUNT4, the Diagnostic and Statistical Manual of Mental Disorders, fifth edition (DSM-5) was used to determine cognitive function, and the procedure has been described in Gjøra et al. [25]. Gait speed was measured with a two time four-metre walk test. The mean speed, in metres per second (m/s), was categorised into > 1.0 m/s, 1.0–0.8 m/s, < 0.8 m/s, and not able to do the test. A gait speed slower than 1.0 m/s, has been associated with increased mortality and adverse health outcomes [26]. An “unknown” category was included in variables where participants had missing observations.

### Statistical analysis

Results are shown for the total study sample and stratified by living situation, sex, and age groups. For individual ROAG-J items (proportions, %) and the ROAG-J total score (median with interquartile range (IQR)) crude results are shown. Prevalence estimates for oral health problems, dental status, and full or partial dentures were further standardised with 95% confidence intervals (CI). Standardisation was applied as described in Gjøra et al*.* [27], where inverse probability weighing (IPW) with adjustments were used to take the invited non-participants in HUNT4 Trondheim 70 + and living situation into account. The weights we used were derived from a logistic regression model where participation was regressed against sex, age groups, and highest attained education based on data for the invited population, obtained from Statistics Norway. Adjustments were further applied to account for the high recruitment of nursing home residents (13.3%) relative to the approximate proportion of long-term nursing home residents ≥ 70 years of age (6.3%) recorded in Trondheim Municipality as of 1 st of January, 2019. This proportion was calculated by the authors based on data from Statbank by Statistics Norway [28] and reference data provided by Trondheim Municipality. Hence, the weight for nursing home residents was multiplied by 0.063/0.133, and (1–0.063)/(1–0.133) for community dwellers. Missing at random was assumed when IPW was applied using the Stata command ‘svy’.

For the ROAG-J total score-results, the Wilcoxon rank sum test was used to test for differences between sexes and living situations, and the Kruskal–Wallis test for differences between age groups. For standardised prevalences, the following analyses were used: for oral health problems, severe oral health problems, and full or partial dentures, differences between sexes and living situation were assessed using the Chi^2^ test. Poison regression was used to assess trend across age groups. For dental status, differences between sexes and between living situations were assessed using the Chi^2^ test, and trend across age groups was assessed using ordered logistic regression. Results were regarded as statistically significant at a two-sided *p* < 0.05. All statistical analyses were performed using Stata Statistical Software: Release 18 (College Station, TX: StataCorp LLC).

## Results

### General characteristics

General characteristics of the study sample are shown in Table 1. Most of the 1562 participants were community dwellers (86.7%), and there were more women (57.6%) than men. The mean age was 78.5 years (SD 7.0), with more women in the oldest age group. Compared to the men, more women lived in nursing homes, had primary education, were widowed, had never smoked, had poor self-perceived health, a BMI < 22 kg/m^2^, dementia, and a gait speed < 0.8 m/s (Table 1). Supplementary Table 1 shows characteristics of the study population stratified by the test locations field station, home visit, and nursing homes. Participants at the field station were on average younger than the participants at the other test locations, and 92.8% of nursing home residents had dementia (Supplementary Table 1, Additional file 1).

**Table 1:** Characteristics of study sample (*n* = 1562)

	**Total** ***n* = 1562**	**Sex**	
		**Women** ***n* = 899 (57.6%)**	**Men** ***n* = 663 (42.4%)**
Age			
Mean (SD)	78.5 (7.0)	79.5 (7.4)	77.2 (6.0)
Age groups, n (%)			
70–74	662 (42.4)	353 (39.3)	309 (46.6)
75–79	362 (23.2)	184 (20.5)	178 (26.9)
80–84	225 (14.4)	139 (15.5)	86 (13.0)
85 +	313 (20.0)	223 (24.8)	90 (13.6)
Living situation, n (%)			
Community dwellers^a^	1354 (86.7)	745 (82.9)	609 (91.9)
Field station	1192 (76.3)	639 (71.1)	553 (83.4)
Home visit	162 (10.4)	106 (11.8)	56 (8.5)
Nursing home residents	208 (13.3)	154 (17.1)	54 (8.1)
Education, n (%)			
Primary (≤ 10 years)	253 (16.2)	195 (21.7)	58 (8.8)
Secondary (11–13 years)	713 (45.7)	426 (47.4)	287 (43.3)
College/University (≥ 14 years)	596 (38.2)	278 (30.9)	318 (48.0)
Marital status, n (%)			
Unmarried	69 (4.4)	41 (4.6)	28 (4.2)
Married	784 (50.2)	335 (37.3)	449 (67.7)
Widow(er)	442 (28.3)	341 (37.9)	101 (15.2)
Separated/Divorced	210 (13.4)	134 (14.9)	76 (11.5)
Unknown	57 (3.7)	48 (5.3)	9 (1.4)
Smoking habits, n (%)			
Never smoker	530 (33.9)	336 (37.4)	194 (29.3)
Previous smoker	700 (44.8)	350 (38.9)	350 (52.8)
Current smoker	72 (4.6)	44 (4.9)	28 (4.2)
Unknown	260 (16.7)	169 (18.8)	91 (13.7)
Self-perceived health, n (%)			
Good	1007 (64.5)	529 (58.8)	478 (72.1)
Poor	284 (18.2)	195 (21.7)	89 (13.4)
Unknown	271 (17.4)	175 (19.5)	96 (14.5)
BMI (kg/m^2^)			
Mean (SD)	26.6 (4.4)	26.3 (4.7)	27.0 (4.0)
Categories, n (%)			
≥ 22 kg/m^2^	1266 (81.1)	681 (75.8)	585 (88.2)
< 22 kg/m^2^	172 (11.0)	132 (14.7)	40 (6.0)
Unknown, n (%)	124 (7.9)	86 (9.6)	38 (5.7)
Cognitive function, n (%)			
No cognitive impairment	730 (46.7)	395 (43.9)	335 (50.5)
MCI	505 (32.3)	281 (31.3)	224 (33.8)
Dementia	307 (19.7)	211 (23.5)	96 (14.5)
Unknown^b^	20 (1.3)	12 (1.3)	8 (1.2)
Gait speed, n (%)			
> 1 m/s	654 (41.9)	346 (38.5)	308 (46.5)
1–0.8 m/s	338 (21.6)	189 (21.0)	149 (22.5)
< 0.8 m/s	424 (27.1)	276 (30.7)	148 (22.3)
Not able to do test	110 (7.0)	70 (7.8)	40 (6.0)
Unknown	36 (2.3)	18 (2.0)	18 (2.7)

*BMI* Body mass index, *MCI* mild cognitive impairment, *m/s* meters per second, *SD* standard deviation

^a^Combines the categories “Field station” and “Home visit”

^b^Other cause of cognitive impairment or not able to diagnose

### Prevalence of oral health problems

**Fig. 2:**
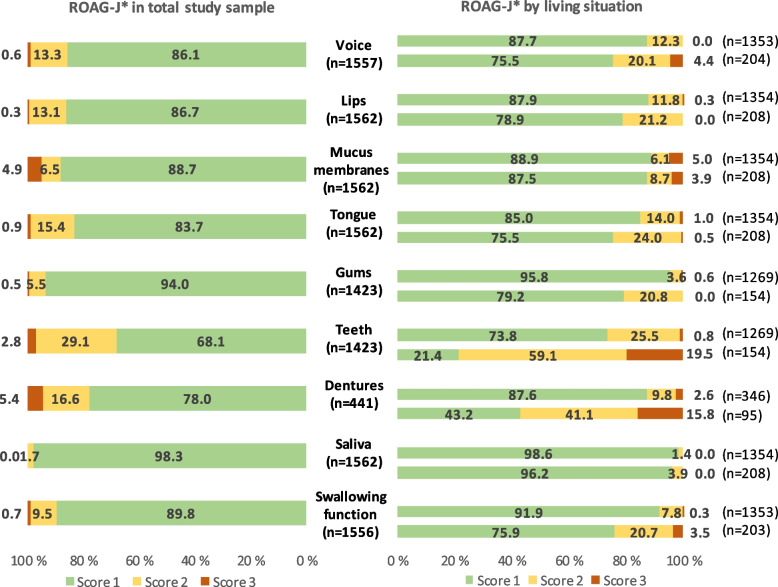
Distribution (%) of scores 1-3 for all ROAG-J items. ROAG-J: Revised Oral Assessment Guide – Jönköping. Left side of bar chart: distribution in the total study sample. Right side of bar chart: distribution stratified by living situation. Each item has two bars: upper bar shows proportions for community dwellers and lower bar show proportions for nursing home residents. Community dwellers include participants from the test locations “Field station” and “Home visit”. *Score 0 excluded for the items voice (*n* = 5), gums (*n* = 139), teeth (*n* = 139), dentures (*n* = 1121), and swallowing function (*n* = 6). Score 1: Normal/healthy condition. Score 2: Moderate oral health problem. Score 3: Severe oral health problem

Figure 2 shows the distribution of scores for each ROAG-J item in the study sample. It was more common to have moderate than severe oral health problems. In the total study sample, the three most common oral health problems (score 2 and 3 combined) were teeth (31.9%), dentures (22.0%), and tongue (16.3%). For severe oral health problems (score 3), the three most common were dentures (5.4%), mucus membranes (4.9%), and teeth (2.8%). Compared to community dwellers, nursing home residents had larger proportions of score 2 and 3 for most items. The difference was especially apparent for teeth (78.6% vs. 26.3%) and dentures (56.9% vs. 12.4%).

Table 2 shows unweighted and standardised prevalences of oral health problems. In the total study sample, the unweighted ROAG-J total score had a range of 9–19 with a median of 10 (IQR 9–11). Nursing home residents had the highest median total score of 11 (IQR 10–12.5). Standardisation altered the prevalences slightly. The standardised prevalence of oral health problems was 56.1% (95% CI 53.5–58.7) and 9.4% (95% CI 8.0–10.9) for severe oral health problems. More men than women had oral health problems, however no difference between the sexes was found for severe oral health problems. There was also a trend across age groups, with an increasing number of participants having oral health problems and severe oral health problems per increase in age group, with those ≥ 85 years of age having the highest prevalence of problems. More nursing home residents compared to community dwellers had oral health problems (85.5% vs. 53.8%) and severe oral health problems (27.0% vs. 8.0%). In addition, among community dwellers, the prevalence of oral health problems was higher among participants examined in their own home compared to participants examined at the field station (63.9% vs. 52.2%).

**Table 2:** Unweighted and standardised prevalences of oral health problems (ROAG-J) (*n* = 1562)

		**ROAG-J total score**^**a**^ **Unweighted**		**Oral health problem**^**b**^		**Severe oral health problem**^**c**^	
	**n**	**Median**	**IQR**	**Unweighted** **n**	**Standardised** **% [95% CI]**	**Unweighted** **n**	**Standardised** **% [95% CI]**
Total	1562	10	9–11	888	56.1 [53.5, 58.7]	167	9.4 [8.0, 10.9]
Sex							
Women	899	10	9–11	485	52.2 [48.7, 55.6]	94	9.3 [7.5, 11.4]
Men	663	10	9–11	403	61.2 [57.2, 65.0]	73	9.4 [7.4, 11.9]
*P*-value		0.04			< 0.001		0.92
Age groups							
70–74	662	9	9–11	320	49.0 [45.1, 53.0]	56	8.3 [6.4, 10.8]
75–79	362	10	9–11	200	55.4 [50.0, 60.7]	34	8.6 [6.1, 12.0]
80–84	225	10	9–11	124	53.8 [46.9, 60.6]	21	8.3 [5.4, 12.7]
85 +	313	10	10–12	244	75.4 [69.7, 80.4]	56	13.7 [10.3, 18.0]
*P*-value		< 0.001			< 0.001		0.03
Living situation							
Community dwellers^d^	1354	10	9–11	710	53.8 [51.1, 56.6]	109	8.0 [6.6, 9.6]
Field station	1192	10	9–11	605	52.2 [49.2, 55.1]	93	7.8 [6.4, 9.5]
Home visit	162	10	9–11	105	63.9 [55.9, 71.2]	16	9.2 [5.6, 14.7]
Nursing home residents	208	11	10–12.5	178	85.5 [79.8, 89.8]	58	27.0 [21.3, 33.5]
*P*-value^e^		< 0.001			< 0.001		< 0.001

*CI* confidence interval, *IQR* Interquartile range, *ROAG-J* Revised Oral Assessment Guide-Jönköping

^a^The score for each ROAG-J item is summated, producing a scale with the possible range of 9–27

^b^At least one oral health problem (score 2 or score 3) in any of the nine ROAG-J items

^c^At least one severe oral health problem (score 3) in any of the nine ROAG-J items

^d^Combines participants from the test locations “Field station” (*n* = 1192) and “Home visit” (*n* = 162)

^e^Statistical analyses for differences between groups were performed between “Community dwellers” and “Nursing home residents”

### Dental status and full or partial dentures

Table 3 presents unweighted and standardised prevalences for dental status and denture use. The prevalence estimates were slightly altered by standardisation. Eighty percent had more than six teeth in both jaws, 10.8% had six or fewer teeth in one or both jaws, and 9.2% were edentulous. In total, 29.6% (95% CI 27.2–32.1) used full or partial dentures. More women than men were edentulous (11.7% vs. 6.0%) and used full or partial dentures (32.3% vs. 26.2%). There was also a trend across age groups, with an increasing number of participants having fewer teeth and edentulism and using dentures per increase in age group. Compared to community dwellers, more nursing home residents had fewer teeth (28.5% vs. 9.4%), were edentulous (29.1% vs. 7.7%), and used dentures (49.5% vs. 28.0%). Participants examined in their own home had higher prevalences of fewer teeth, edentulism, and denture use compared to those examined at the field station.

**Table 3:** Unweighted and standardised prevalences for dental status and denture use (*n* = 1548)

		** > 6 teeth in both jaws**		** ≤ 6 teeth in one or both jaws**		**Edentulous**		**Full or partial dentures**	
	**n**	**Unweighted** **n**	**Standardised** **% [95%CI]**	**Unweighted** **n**	**Standardised** **% [95%CI]**	**Unweighted** **n**	**Standardised** **% [95%CI]**	**Unweighted** **n**	**Standardised** **% [95%CI]**
Total	1548^a^	1243	80.0 [77.7, 82.0]	166	10.8 [9.2, 12.6]	139	9.2 [7.8, 10.9]	435	29.6 [27.2, 32.1]
Sex									
Women	890	690	77.5 [74.4, 80.3]	97	10.8 [8.8, 13.2]	103	11.7 [9.6, 14.2]	275	32.3 [29.1, 35.7]
Men	658	553	83.2 [79.8, 86.1]	69	10.8 [8.5, 13.7]	36	6.0 [4.3, 8.4]	160	26.2 [22.7, 30.0]
*P*-value		0.002							0.02
Age groups									
70–74	657	600	90.3 [87.6, 92.5]	34	5.6 [4.0, 7.8]	23	4.1 [2.7, 6.2]	133	21.5 [18.3, 25.0]
75–79	356	307	85.1 [80.6, 88.7]	33	10.2 [7.3, 14.3]	16	4.7 [2.8, 7.7]	84	26.0 [21.4, 31.3]
80–84	223	179	78.9 [72.4, 84.2]	29	13.1 [9.0, 18.7]	15	8.0 [4.7, 13.1]	65	31.6 [25.4, 38.6]
85 +	312	157	49.7 [43.6, 55.8]	70	21.6 [17.0, 27.1]	85	28.7 [23.4, 34.6]	153	51.6 [45.5, 57.7]
P for trend		< 0.001							< 0.001
Living situation									
Community dwellers^b^	1342	1148	82.9 [80.5, 85.0]	109	9.4 [7.8, 11.3]	85	7.7 [6.2, 9.4]	342	28.0 [25.5, 30.7]
Field station	1182	1067	88.8 [86.6, 90.6]	79	7.7 [6.2, 9.6]	36	3.5 [2.5, 4.9]	256	23.3 [20.8, 26.0]
Home visit	160	81	47.1 [39.2, 55.0]	30	19.9 [14.2, 27.3]	49	33.0 [25.9, 41.0]	86	56.9 [48.9, 64.5]
Nursing home residents	206	95	42.4 [35.6, 49.4]	57	28.5 [22.6, 35.4]	54	29.1 [23.0, 36.1]	93	49.5 [42.5, 56.6]
*P*-value^c^		< 0.001							< 0.001

*CI* confidence interval

^a^14 participants were excluded due to missing information about number of teeth in one or both jaws

^b^Combines participants from the test locations “Field station” (*n* = 1182) and “Home visit” (*n* = 160)

^c^Statistical analyses for differences between groups were performed between “Community dwellers” and “Nursing home residents”

## Discussion

In this Norwegian older adult population, 56.1% had at least one oral health problem, either moderate or severe, and 9.4% had at least one severe problem indicating the need for treatment by a dentist or physician. Edentulism was found in 9.2%, and 29.6% used full or partial dentures. Men had a higher prevalence of oral health problems, but more women were edentulous and used dentures. Those ≥ 85 years of age and nursing home residents had the highest prevalence of oral health problems, edentulism, and dentures. Moreover, community dwellers examined in their own home showed higher prevalences for most oral health measures compared to those examined at the field station.

To the best of our knowledge, the current study is the first to use ROAG-J data to provide standardised estimates of oral health problems in both community dwellers and nursing home residents. The use of ROAG(-J) has been limited to samples of nursing home residents and adults admitted to hospital wards or other care facilities [29–33]. Studies investigating a similar diverse older adult population using other comprehensive methods to assess oral health in Europe are however also scarce. Still, our results do align with studies investigating community dwellers, individuals in care facilities and care-dependent populations independently, showing that oral health problems (self-reported or clinically assessed) are prevalent across the older adult population [31, 34–40].

We found a prevalence of edentulism of 9.2%, which is within the range of 2–18% found in neighbouring Scandinavian countries in 2014 and 2017 [4, 41]. However, in care facilities and nursing homes, the estimates are higher, ranging from 19–23% in studies using ROAG(-J) in Sweden [29, 31, 33]. Population estimates of full or partial denture use among older adults in Scandinavian countries are however scarce. In Sweden, two studies found a prevalence of 3–4% among 20–89-year-old community dwellers [7, 42]. In nursing homes and other care facilities, the corresponding estimates for the use of full or partial dentures range from 35–40% in studies using ROAG(-J) [29, 31, 33]. That dental status and denture use possibly differ between living situations aligns with our findings, although our estimates were higher. It is likely that the gap between estimates can be explained by differences in participant characteristics such as age and cognitive function, but also geographical differences. It is also possible that differences in accessibility to oral health care and statutory coverage between the Scandinavian countries could be an explanatory factor [43].

In the current study, the most pronounced differences between the sexes were related to the total proportion with oral health problems and the dentition. The reason for this might be multifactorial, including possible differences in oral hygiene and help-seeking behaviour [44]. Older women have shown to be more concerned about the appearance of their teeth and mouth [45], which could suggest an influence by socially constructed gender norms. Similar higher prevalences of the total proportion of oral health problems among men have also been found among nursing home residents in studies using ROAG-J [31]. Also, the prevalence of individual problems has been shown to differ between sexes [33]. We further found that more women were edentulous and used dentures. Although women have been found to have a higher prevalence of edentulism [10], there are also some population studies suggesting that edentulism and denture use might be similar between the sexes [5, 41].

In our study, increasing age was followed by higher prevalences of oral health problems, fewer teeth and edentulism, and denture use. This aligns with previous studies on community dwelling adults showing worse dental health with increasing age [5, 9, 34, 46]. However, among older adults in need of care, some studies suggest there might not be an age-related trend for oral health problems and oral hygiene [29, 31, 37, 39]. Although chronological age is important in the sense of exposure time and accumulation of potential oral problems, it should be considered in relation to general health. In Bellander et al*.* [31], a larger proportion of oral health problems was found in their youngest age group (65–74 years) of nursing home residents compared to the older age groups. This was explained by possible long-term illness and nursing home residence before the age of 65 in this group [31]. The access to residential care and home help have become restricted to those with the highest needs in the Nordic countries [47]. In our study, almost all nursing home residents had dementia, and it is possible that this could explain the high prevalence of oral health problems (85.5%) in this group. In comparison, in Swedish care facilities, estimates of oral health problems have ranged from 42% in nursing homes [31] to 77% in short-term care [29].

Our findings show that many older adults, both community dwellers and nursing home residents, have suboptimal oral health. Additionally, our results also indicate that within the group of community dwellers, some have poorer oral health than others. This aligns with studies showing similar poor oral health conditions among vulnerable home dwelling older adults and nursing home residents [37, 39]. Oral health problems, dental status, and dentures have been linked to quality of life, the risk of malnutrition, and all-cause mortality in older adults [48–50]. To prevent oral health problems, sufficient oral hygiene throughout life is crucial [51]. This can however be challenging as many factors may influence personal oral care, e.g. physical and cognitive function, emotions, motivation and attitude, access to proper utilities, and help [52]. Care-dependent older adults are especially vulnerable as they might not be able to perform adequate oral self-care. This requires that health care personnel or relatives providing the help have the knowledge and resources needed. Even though there is willingness from care providers to perform oral care, they might be restricted by accessibility, time, available resources and knowledge, in addition to the health and wishes of the older persons [53, 54]. This further underlines the importance of oral health awareness and the need for preventive oral care across the older population.

Our study has both strengths and limitations. This study is one of few to provide standardised estimates of oral health measures in a population of adults aged 70 years and older. The data collected in HUNT4 Trondheim 70 + is unique as it includes the oldest in a population, both independent and care needing adults. Among the participants, the ROAG-J assessment was completed in 90% across all test locations. As a result, we had a large and heterogeneous sample of older adults of both community dwellers and nursing home residents. Even so, readers should be aware of the possibility of selection bias in our study due to the low participation in Trondheim 70 + (34%). The population in the HUNT4 survey has been described as generalisable to populations of European ancestry [17]. Furthermore, the catchment area of HUNT4 Trondheim 70 + was limited to districts of a Norwegian city. Thus, our results might have limited generalisability to populations with other ancestries, rural populations, and might display regional traits. It is also worth noting that regional differences in tooth loss and denture use among older adults have been observed in Norway [55]. Additionally, even though most items in ROAG have shown good accuracy, some items, especially saliva, has shown low sensitivity [22]. In HUNT4 Trondheim 70 +, the item saliva was assessed using a toothbrush, and the ROAG-J assessment was conducted by multiple health care personnel. This could have introduced measurement error and inter-assessment variation in the results even though training was provided. Underestimation of problems related to saliva is therefore probable in our study.

## Conclusion

In this Norwegian older adult population aged ≥ 70 years, 56% had at least one oral health problem. The prevalences of oral health problems, few teeth and edentulism, and full or partial dentures were highest among the oldest and nursing home residents. We find reason to propose raised awareness regarding preventive oral health care across the older adult population, including primary health care.

## Supplementary Information

Additional file 1: Supplementary Table 1. characteristics of the study sample stratified by test location.

## Data Availability

The Trøndelag Health Study (HUNT) has invited persons aged 13—100 years to four surveys between 1984 and 2019. Comprehensive data from more than 140,000 persons having participated at least once and biological material from 78,000 persons are collected. The data are stored in HUNT databank and biological material in HUNT biobank. HUNT Research Centre has permission from the Norwegian Data Inspectorate to store and handle these data. The key identification in the data base is the personal identification number given to all Norwegians at birth or immigration, whilst de-identified data are sent to researchers upon approval of a research protocol by the Regional Ethical Committee and HUNT Research Centre. To protect participants’ privacy, HUNT Research Centre aims to limit storage of data outside HUNT databank, and cannot deposit data in open repositories. HUNT databank has precise information on all data exported to different projects and are able to reproduce these on request. There are no restrictions regarding data export given approval of applications to HUNT Research Centre. For more information see: http://www.ntnu.edu/hunt/data.

## Abbreviations

BMI: Body mass index
CI: Confidence interval
DSM-5: The Diagnostic and Statistical Manual of Mental Disorders, fifth edition
HUNT: The Trøndelag Health Study
IPW: Inverse probability weighting
IQR: Interquartile range
Kg/m^2^: Kilograms over meters squared
MCI: Mild cognitive impairment
M/s: Meters per second
NTNU: Norwegian University of Science and Technology
OAG: Oral Assessment Guide
ROAG: Revised Oral Assessment Guide
ROAG-J: Revised Oral Assessment Guide- Jönköping
SD: Standard deviation
