# Supplementary material for: Prevalence of oral health problems in a Norwegian older adult population: The HUNT Study
Source: BMC Oral Health. 2025 Sep 30;25:1505. doi: 10.1186/s12903-025-06683-y (PMC12482855; doi:10.1186/s12903-025-06683-y)
Supplement: Supplementary file 1 — Additional file 1: Supplementary Table 1. characteristics of the study sample stratified by test location. [file 12903_2025_6683_MOESM1_ESM.docx]

Supplementary table 1: Characteristics of the study sample by test location (n=1562)

|  | **Field station**  **n=1192 (76.3%)** | **Home visit**  **n=162 (10.4%)** | **Nursing home**  **n=208 (13.3%)** |
| --- | --- | --- | --- |
| **Sex, n (%)** |  |  |  |
| Women | 639 (53.6) | 106 (65.4) | 154 (74.0) |
| Men | 553 (46.4) | 56 (34.6) | 54 (26.0) |
| **Age** |  |  |  |
| Mean (SD) | 76.1 (4.8) | 84.0 (7.0) | 87.8 (6.9) |
| Age groups, n (%) |  |  |  |
| 70-74 | 624 (52.4) | 26 (16.1) | 12 (5.8) |
| 75-79 | 327 (27.4) | 20 (12.4) | 15 (7.2) |
| 80-84 | 156 (13.1) | 34 (21.0) | 35 (16.8) |
| 85+ | 85 (7.1) | 82 (50.6) | 146 (70.2) |
| **Education, n (%)** |  |  |  |
| Primary (≤10 years) | 113 (9.5) | 59 (36.4) | 81 (38.9) |
| Secondary (11-13 years) | 548 (46.0) | 75 (46.3) | 90 (43.3) |
| College/University (≥14 years) | 531 (44.6) | 28 (17.3) | 37 (17.8) |
| **Marital status, n (%)** |  |  |  |
| Unmarried | 51 (4.3) | 9 (5.6) | 9 (4.3) |
| Married | 721 (60.5) | 38 (23.5) | 25 (12.0) |
| Widow(er) | 262 (22.0) | 84 (51.9) | 96 (46.2) |
| Separated/Divorced | 157 (13.2) | 31 (19.1) | 22 (10.6) |
| Unknown | 1 (0.1) | 0 (0.0) | 56 (26.9) |
| **Smoking habits, n (%)** |  |  |  |
| Never smoker | 458 (38.4) | 35 (21.6) | 37 (17.8) |
| Previous smoker | 601 (50.4) | 43 (26.5) | 56 (26.9) |
| Current smoker | 66 (5.5) | 4 (2.5) | 2 (1.0) |
| Unknown | 67 (5.6) | 80 (49.4) | 113 (54.3) |
| **Self-perceived health, n (%)** |  |  |  |
| Good | 935 (78.4) | 38 (23.5) | 34 (16.4) |
| Poor | 178 (14.9) | 46 (28.4) | 60 (28.9) |
| Unknown | 79 (6.6) | 78 (48.2) | 114 (54.8) |
| **BMI (kg/m^2^)** |  |  |  |
| Mean (SD) | 26.6 (4.2) | 27.0 (5.5) | 26.2 (5.5) |
| Categories, n (%) |  |  |  |
| ≥22 kg/m^2^ | 1053 (88.3) | 119 (73.5) | 94 (45.2) |
| <22 kg/m^2^ | 130 (10.9) | 21 (13.0) | 21 (10.1) |
| Unknown, n (%) | 9 (0.8) | 22 (13.6) | 93 (44.7) |
| **Cognitive function, n (%)** |  |  |  |
| No cognitive impairment | 683 (57.3) | 42 (25.9) | 5 (2.4) |
| MCI | 432 (36.2) | 64 (39.5) | 9 (4.3) |
| Dementia | 73 (6.1) | 41 (25.3) | 193 (92.8) |
| Unknown^a^ | 4 (0.3) | 15 (9.3) | 1 (0.5) |
| **Gait speed, n (%)** |  |  |  |
| >1 m/s | 649 (54.5) | 3 (1.9) | 2 (1.0) |
| 1-0.8 m/s | 311 (26.1) | 23 (14.2) | 4 (1.9) |
| <0.8 m/s | 205 (17.2) | 99 (61.1) | 120 (57.7) |
| Not able to do test | 2 (0.2) | 33 (20.4) | 75 (36.1) |
| Unknown | 25 (2.1) | 4 (2.5) | 7 (3.4) |

BMI: Body mass index, MCI: mild cognitive impairment, m/s: meters per second, SD: standard deviation

^a^ Other cause of cognitive impairment or not able to diagnose.
